# Dissecting Human Embryogenesis With Blastoids: Insights Into Lineage Specification and Early Development

**DOI:** 10.1002/rmb2.70059

**Published:** 2026-05-09

**Authors:** Hiroo Sasaki, Ayaka Yanagida

**Affiliations:** ^1^ Department of Veterinary Anatomy The University of Tokyo Tokyo Japan; ^2^ Stem Cell Therapy Laboratory, Advanced Research Institute, Institute of Science Tokyo Tokyo Japan

**Keywords:** blastocyst, cell differentiation, cell lineage, embryo implantation, pluripotent stem cells

## Abstract

**Background:**

Understanding human embryogenesis is a fundamental goal in developmental biology and reproductive medicine, but direct investigation of human embryos is limited by ethical and technical constraints. Stem cell–based blastocyst models have emerged as alternative systems to recapitulate key features of early human development in vitro.

**Methods:**

We reviewed the literature on human stem cell–based embryo models, with a focus on blastoids. Relevant studies and review articles were identified through PubMed, covering blastoid generation strategies, model diversity, and insights into early lineage specification derived from blastoids and pluripotent stem cell studies.

**Main Findings:**

Blastoids provide a versatile platform to model key aspects of human blastocyst development, including lineage specification, embryonic–extraembryonic interactions, and early morphogenesis. Recent studies demonstrate their utility in modeling implantation‐related processes, embryo–maternal interactions, and responses to genetic, environmental, and pharmacological perturbations. However, current systems remain limited by incomplete lineage representation and restricted capacity to model post‐implantation‐like development. Ongoing advances in stem cell biology and culture systems are expected to improve their fidelity and developmental potential.

**Conclusion:**

Blastoids represent a promising approach for studying early human embryogenesis and are expected to expand their applications in reproductive biology and medicine.

## 1 Introduction

Understanding early human embryogenesis is fundamental for improving assisted reproductive technologies and for elucidating the origin of congenital disorders. Following fertilization, the zygote undergoes a series of cleavage divisions, progressing through the two‐cell, four‐cell, and eight‐cell stages before forming a morula and eventually developing into a blastocyst. In mice, blastocyst formation occurs approximately 3.5 days after fertilization (embryonic day [E] 3.5), followed by implantation into the uterus by E4.5. In humans, blastocysts form around Day 5 after fertilization, and implantation begins at approximately Day 7 [1, 2]. The mature blastocyst consists of three major cell lineages: the trophectoderm, which gives rise to the placenta; the hypoblast, which contributes to the yolk sac; and the epiblast, which serves as the founder of the embryo proper. Proper establishment and coordinated differentiation of these lineages are essential for early embryonic development. However, because early developmental processes occur within the maternal environment, direct investigation of their underlying molecular mechanisms and cellular dynamics has long remained technically and ethically challenging.

**FIGURE 1:**
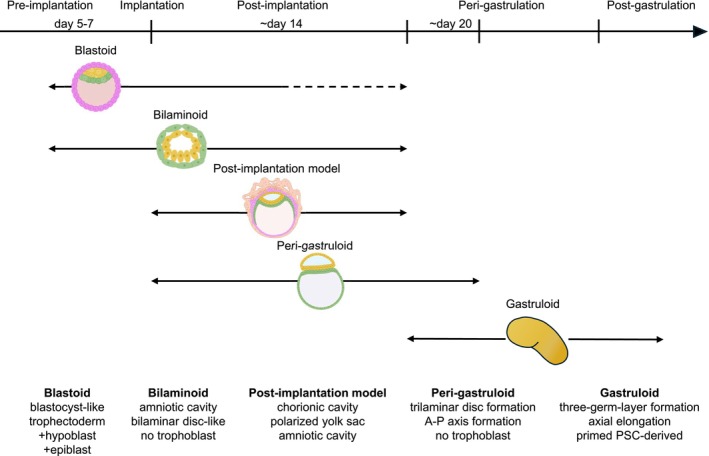
Human stem cell‐based embryo models recapitulating distinct stages of early embryogenesis. Arrows indicate the developmental range covered by each model. Despite recent advances, blastoid models remain limited in their capacity to recapitulate post‐implantation development.

Major advances in stem cell biology have significantly enhanced the study of early mammalian development. The derivation of mouse embryonic stem cells in 1981 [3, 4] and human embryonic stem cells in 1998 [5] provided in vitro systems that capture key properties of the epiblast. Embryonic stem cells (ESCs) are pluripotent cell lines established from the epiblast of blastocysts. They closely resemble their in vivo counterparts, the epiblast, while possessing self‐renewal capacity and the ability to differentiate into all embryonic lineages. Therefore, ESCs enabled developmental processes to be partially recapitulated in vitro and have greatly advanced understanding of embryogenesis. More recently, stem cell lines corresponding to the other blastocyst lineages, including primitive endoderm (the equivalent of hypoblast in humans) and trophectoderm, have also been established [6, 7, 8]. Together, these advances have expanded experimental access not only to embryo proper but also to extraembryonic tissues such as the yolk sac and placenta. Building on these advances, studies have sought to reconstruct key aspects of embryogenesis using stem cells. Initial approaches began to reproduce differentiation toward specific cell lineages in vitro using stem cells, typically using two‐dimensional culture systems. These efforts have led to the emergence of recapitulating three‐dimensional structures of more complex tissues, exemplified by organoid systems. More recently, these technologies have further advanced to enable the reconstruction of organized structures that capture key features of the early embryos. Stem cell‐derived three‐dimensional structures that recapitulate key features of early embryos are referred to as stem cell‐based embryo models (SCBEMs). A variety of human embryo models have now been developed to capture distinct stages of early embryogenesis (Figure 1). These include blastoids, which recapitulate aspects of the pre‐implantation blastocyst [9, 10, 11]; post‐implantation embryo models that mimic developmental stages up to approximately 14 days after fertilization [12, 13, 14]; peri‐gastruloids, which mimic certain features of post‐implantation embryos around Day 20 but lack trophoblast lineages [15]; bilaminoids, which model the bilaminar embryonic disc but also lack trophoblast lineages [16]; and gastruloids, which aim to recapitulate aspects of post‐gastrulation embryogenesis [17]. Although these models do not fully reproduce the developmental potential or structural complexity of natural human embryos, they have rapidly become powerful experimental systems. In particular, they enable experimental investigation of human embryonic developmental processes that have long been inaccessible to direct investigation, including aspects of implantation and early post‐implantation embryos.

In this Review, we focus on blastoids, SCBEMs that mimic the pre‐implantation blastocyst. We discuss how these systems have advanced understanding of lineage specification and early human development, and examine the extent to which they recapitulate key developmental processes. We also highlight current limitations and future directions for improving their biological fidelity and experimental utility.

## 2 Approaches to Blastoid Generation

**FIGURE 2:**
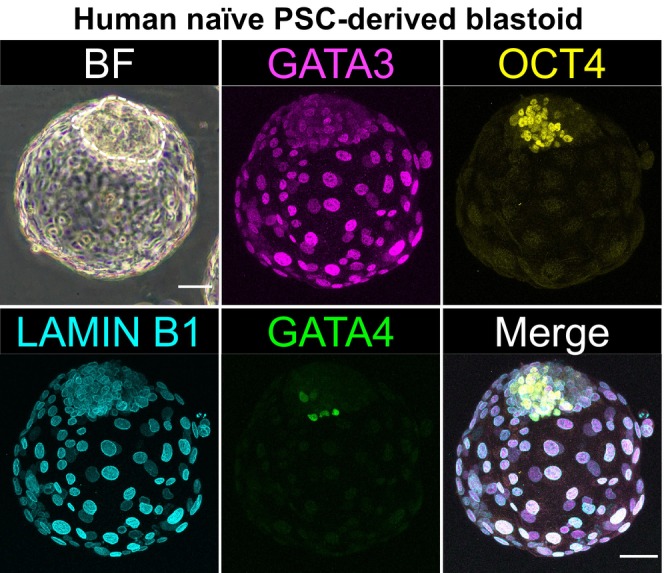
Representative image of human naïve PSC‐derived blastoid showing three lineages. Phase‐contrast and immunofluorescence images of a human blastoid derived from naïve PSCs, showing lineage‐specific marker expression: GATA3 (trophectoderm), OCT4 (epiblast), and GATA4 (hypoblast). The ICM‐like cell mass is outlined with a dotted line. The authors for this review prepared this figure. Scale bars, 50 μm.

**FIGURE 3:**
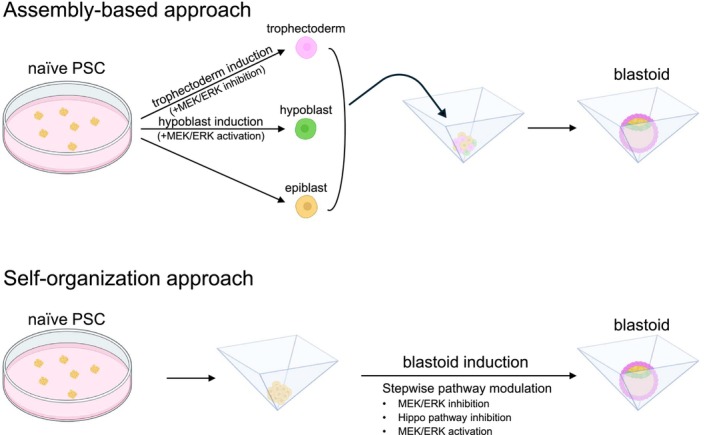
Strategies for blastoid generation from naïve PSCs. Schematic comparison of assembly‐based and self‐organization approaches for blastoid generation from naïve PSCs. Key signaling pathways involved in lineage induction are indicated.

To mimic the blastocyst, it is essential to reproduce the three cell lineages that constitute it: the epiblast, hypoblast, and trophectoderm (Figure 2). Conceptually, current strategies for generating blastoids can be broadly categorized into two types: assembly‐based approaches and self‐organization approaches (Figure 3). In assembly‐based approaches, individual blastocyst lineages are generated separately and subsequently combined to form blastocyst‐like structures. In contrast, self‐organization approaches rely on the differentiation potential of pluripotent stem cells (PSCs), allowing multiple blastocyst lineages to emerge from a single cell population. Although the starting cell types and culture conditions vary among studies, both approaches rely on cell–cell interactions to reconstruct blastocyst‐like structures.

### 2.1 Assembly‐Based Approaches

Assembly‐based strategies involve generating the major blastocyst lineages in vitro through directed differentiation of PSCs, followed by mixing these cell populations at defined ratios and timings to promote the formation of blastocyst‐like structures. A key advantage of this approach is that individual lineages can be manipulated and controlled independently.

The first blastoids were reported in mouse studies using this strategy. In 2018, Rivron et al. generated blastocyst‐like structures by combining ESCs with trophoblast stem cells (TSCs), allowing the cells to self‐aggregate and organize into structures resembling blastocysts [18]. Assembly‐based approaches have been widely used in mice and remain a predominant strategy in part because mouse ESCs cannot readily be induced directly into the trophectoderm lineage without genetic manipulation.

In contrast, assembly‐based approaches for human blastoids remain relatively limited, partly due to the lack of well‐established stem cell lines corresponding to hypoblast and trophectoderm lineages. One example of an assembly‐based approach used extended pluripotent stem cells (EPSCs) [19] combined with trophectoderm‐like cells derived from EPSC differentiation induced by BMP4 to generate blastocyst‐like structures [20]. As assembly‐based systems continue to be refined, they may enable systematic interrogation of individual blastocyst lineages and their interactions during early development.

### 2.2 Self‐Organization Approaches

Self‐organization strategies rely on the differentiation potential of PSCs, allowing multiple blastocyst lineages to arise from a single cell population. Typically, naïve PSCs or heterogeneous cell states that emerge during somatic cell reprogramming are used as starting materials.

The emergence of human blastoids was closely linked to advances in studies of naïve PSCs. Naïve PSCs correspond to the preimplantation epiblast across species, although their differentiation potentials differ between species. Notably, human naïve PSCs can differentiate into extraembryonic lineages such as trophectoderm and hypoblast under appropriate culture conditions [16, 21, 22, 23], a property that is not readily observed in naïve mouse PSCs without genetic manipulation.

Multiple methods for deriving human naïve PSCs have been reported, each employing distinct derivation and culture conditions [24, 25, 26]. Consequently, protocols for generating blastoids vary depending on the human PSCs used. Consistent with this, the first reports of human blastoids were generated using different starting cell systems. Yu et al. used human naïve PSCs maintained under 5i/L/A conditions (MEK/ERK inhibitor, GSK3 inhibitor, BRAF inhibitor, LCK/SRC inhibitor, ROCK inhibitor, human LIF, Activin A) [25] and developed a two‐step protocol in which cells were first induced toward hypoblast‐like lineage and subsequently toward trophectoderm‐like lineage, or vice versa, to generate blastoids [9]. Yanagida et al. generated blastoids from human naïve PSCs maintained under PXGL conditions (MEK/ERK inhibitor, tankyrase inhibitor, aPKC inhibitor, human LIF) [27] by transiently exposing the cells to trophectoderm‐inducing signals to induce trophectoderm‐like lineage and later hypoblast‐like lineage [11, 21]. Liu et al. reported the generation of blastoids from heterogeneous cell states, arising during somatic cell reprogramming toward induced PSCs [10].

Since these initial reports, refinements in culture conditions, differentiation strategies, and starting cell types continue to be introduced, enabling the generation of models that more faithfully mimic human embryogenesis.

### 2.3 Evaluation and Current Challenges

Accurate evaluation is essential to determine how faithfully blastoids recapitulate natural embryonic development. Evaluation of blastoids requires assessment of their similarity to natural blastocysts at both morphological and molecular levels. Key criteria include the presence and relative proportions of the three blastocyst lineages, their spatial organization, cell numbers and positioning, and their ability to model subsequent developmental stages. Among these evaluation methods, transcriptome analyses have been widely used to assess the similarity between blastoids and natural embryos.

Transcriptomic analyses in the initial reports of human blastoids suggested that these blastoids contained cell populations corresponding to human embryos at approximately Days 5–7 post‐fertilization. However, subsequent re‐analyses have suggested that in some reported blastoids, a subset of cell populations initially interpreted as trophectoderm‐like cells may instead represent amnion‐like cells, which arise after implantation and share partially overlapping gene expression profiles with trophectoderm [28, 29].

The presence of cell types that are not normally found in the natural preimplantation blastocyst may influence the phenotypes and signaling responses observed in blastoid‐based experimental systems. Therefore, rigorous evaluation of blastoid composition and lineage identity is essential for accurately modeling human embryogenesis. In addition, ensuring consistency in blastoid quality across experiments is important for improving experimental reproducibility and enabling future applications in disease modeling.

## 3 Lineage Specification in Blastoid Formation

Blastoid formation relies on the coordinated emergence of the three blastocyst lineages: epiblast, hypoblast, and trophectoderm. During blastoid formation, key features of blastocyst development are recapitulated, including the emergence of trophectoderm‐ and hypoblast‐like cells and the progressive maturation of lineage identities.

Together with advances in naïve PSC studies, blastoid models have not only recapitulated key features of blastocyst lineage specification but have also provided experimental platforms to directly test the cellular dynamics and molecular mechanisms governing lineage specification during early embryogenesis. In this section, we summarize current understanding of lineage specification in the blastocyst and discuss how these mechanisms contribute to blastoid formation.

### 3.1 Epiblast

The epiblast arises from the inner cell mass (ICM) of the blastocyst and gives rise to the embryo proper. In blastoid systems, epiblast‐like cells typically originate from the PSCs used and form the central embryonic compartment of the model.

Understanding epiblast identity in blastoids requires considering its in vivo counterparts. The epiblast exhibits distinct transcriptional and functional states before and after implantation, referred to as naïve and primed pluripotent states, respectively [30]. Furthermore, recent studies have identified an additional pluripotent state, termed the formative state, which represents a transitional phase between naïve and primed pluripotent states [31, 32]. The transition from naïve to formative and subsequently to primed states during embryonic development is important for epiblast cells to acquire multi‐lineage competence, with the formative state representing a key phase during which cells become competent to differentiate into both germline and somatic lineages. This transition is also important for enabling appropriate responses to lineage‐specifying signaling during subsequent developmental progression, particularly in the context of modeling post‐implantation‐like stages from blastoids in vitro.

Although conventional human ESCs are derived from preimplantation blastocysts [5], they resemble post‐implantation epiblast states, similar to mouse epiblast stem cells (EpiSCs) [33, 34], rather than mouse ESCs, which correspond to the preimplantation epiblast [3, 4]. Accordingly, conventional human ESCs are categorized as primed PSCs. In contrast, human naïve PSCs capture features of the preimplantation epiblast [25, 35] and exhibit broader developmental potential. Accordingly, many blastoid protocols use naïve PSCs as a starting population.

**FIGURE 4:**
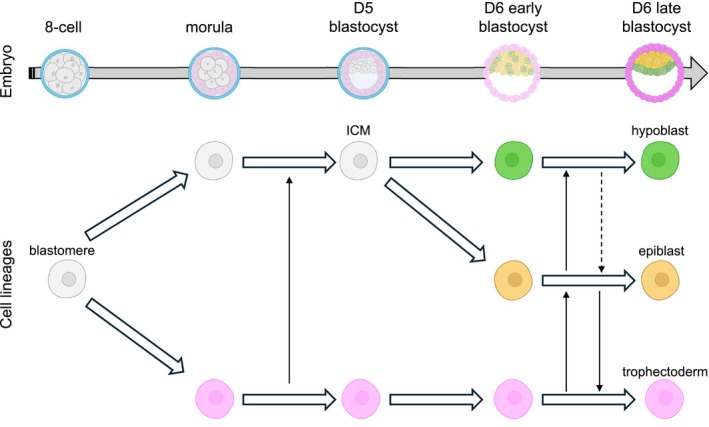
Lineage specification and plasticity during early human embryogenesis. Schematic overview of lineage specification and lineage plasticity during early human embryogenesis. Solid arrows indicate experimentally supported lineage plasticity between cell types, whereas dashed arrows indicate putative plasticity that has not yet been clearly demonstrated. Pale‐colored cells represent precursor states that retain plasticity, whereas darker‐colored cells represent committed cell states with reduced plasticity.

A key property of the preimplantation human epiblast is pluripotency and its lineage plasticity, including the potential to differentiate into extraembryonic lineages under certain conditions [21, 22]. Lineage plasticity is also observed during early embryonic development, although its extent and context differ between species and developmental stages. In mouse embryogenesis, cell fate decisions occur progressively, with the initial distinction between trophectoderm and the ICM at the morula stage, followed by differentiation of the ICM into epiblast and primitive endoderm lineages [36]. Despite this progression, cells retain lineage plasticity for a limited period [37, 38]. Under experimental conditions, ICM cells can contribute to trophectoderm, for example following immunosurgical isolation at the early blastocyst stage [39], or after reintroduction into morula‐stage embryos [38], indicating that ICM cells retain lineage plasticity toward the trophectoderm lineage at early stages, although this potential is lost by mid‐blastocyst stages. As ICM cells begin to differentiate into epiblast and primitive endoderm lineages during the early to mid‐blastocyst stage [40], they still retain the capacity to interconvert between epiblast and primitive endoderm fates [37]. As development proceeds, lineage commitment becomes progressively established, leading to a loss of lineage plasticity by the late blastocyst stage. In human embryos, a similar sequence of cell fate decisions occurs, from the segregation of trophectoderm and ICM to the subsequent differentiation of the ICM into epiblast and hypoblast, as schematically illustrated in Figure 4. However, epiblast plasticity toward extraembryonic lineages persists over a broader developmental window compared to mice. ICM or epiblast cells from late‐stage human blastocysts retain the capacity to differentiate into trophectoderm [21]. Human naïve PSCs, which correspond to the preimplantation epiblast, also exhibit the ability to differentiate into both trophectoderm and hypoblast without genetic manipulation [21, 22], a property that underlies the self‐organization of human blastoids [9, 11].

Despite these advances, current naïve human PSCs do not fully recapitulate the in vivo preimplantation epiblast state, particularly in their epigenetic states at imprinting loci, which may influence developmental competence [41, 42]. Further refinement of naïve PSC culture systems will therefore be important for improving the fidelity of blastoid models and more accurately capturing human embryogenesis.

### 3.2 Hypoblast

The hypoblast arises from the ICM and contributes to yolk sac formation and embryonic support functions, playing a critical role in embryogenesis. Hypoblast defects are associated with reduced implantation potential in human embryos [43]. In blastoid systems, proper specification of the hypoblast lineage is essential for recapitulating blastocyst organization and supporting subsequent developmental processes.

The mechanisms governing hypoblast specification and maturation in human embryos have long remained poorly understood. However, recent studies have begun to clarify these processes. In mouse embryos, lineage segregation within the ICM gives rise to the epiblast and primitive endoderm [40]. However, whether similar lineage segregation occurs in humans remained unclear for many years. Recent single‐cell transcriptomic analyses suggest that hypoblast is specified from the ICM during human preimplantation development [44, 45, 46, 47], consistent with earlier observations from human embryos [48, 49, 50]. These studies have also characterized hypoblast‐specific markers and their temporal dynamics, advancing our understanding of hypoblast specification [51].

In mouse embryos, differentiation of primitive endoderm is strongly dependent on FGF/ERK signaling [52, 53]. Recent studies suggest that FGF/ERK signaling also contributes to hypoblast specification in humans, indicating a broadly conserved role [23, 54]. These signaling mechanisms are also relevant for in vitro systems, including blastoids.

Building on these insights, recent studies have begun to define conditions for inducing hypoblast‐like cells from human naïve PSCs. Overexpression of GATA6 or treatment with defined combinations of signaling factors (including FGF4, BMP4, IL‐6, PDGF‐AA, Wnt/β‐catenin inhibitor, ALK4/5/7 inhibitor, and retinoic acid) can induce hypoblast‐like cells with transcriptional profiles similar to preimplantation hypoblast [16]. In parallel, FGF/ERK signaling has been identified as a key regulator of hypoblast induction from human naïve PSCs and human embryos [23]. Together, these findings highlight a central role for FGF/ERK signaling in hypoblast induction, although additional pathways may also contribute.

In human blastoids, the hypoblast lineage is often underrepresented [11, 55]. In self‐organization approaches, improving culture conditions during blastoid induction may enhance hypoblast formation, whereas in assembly‐based approaches, establishing robust and stable hypoblast‐like stem cells will be critical. Together, these advances will improve the fidelity of blastoids to natural human blastocysts and enhance their utility as experimental models for studying early human development.

### 3.3 Trophectoderm

The trophectoderm forms the outer epithelial layer of the blastocyst and serves as the initial interface between the embryo and the uterus. Following implantation, trophectoderm differentiates into cytotrophoblast, syncytiotrophoblast, and extravillous trophoblast, contributing to placental development and maternal–fetal interactions [56, 57]. In blastoid systems, proper specification of the trophectoderm lineage is essential for blastoid formation and modeling implantation‐related processes.

The mechanisms underlying trophectoderm specification have been extensively studied in mouse embryos [58]. Following compaction, outer blastomeres acquire apicobasal polarity and give rise to trophectoderm, whereas inner cells form the ICM [59]. Genetic studies identified essential roles for the transcription factor Cdx2 [60] and its upstream regulator Tead4 [61] in trophectoderm formation. Subsequent work revealed that the Hippo–Yap/Tead pathway plays a central role in this process: reduced Hippo signaling in outer cells leads to nuclear localization of Yap, which activates trophectoderm‐specific transcriptional programs through Tead4 [62]. This pathway is regulated by cell polarity, such that apicobasal polarity in outer cells suppresses Hippo signaling and promotes trophectoderm fate. Recent analyses of human embryos indicate that this Hippo–Yap/Tead regulatory axis is conserved in humans, suggesting shared principles of trophectoderm specification across species [63, 64].

In humans, direct investigation of preimplantation trophectoderm specification has long been limited by ethical and technical constraints and the scarcity of embryo samples. To model trophectoderm specification in vitro, studies of trophectoderm induction from PSCs have provided important insights and revealed species‐specific differences. In mouse PSCs, overexpression of Cdx2 is required to drive differentiation toward the trophectoderm lineage [65], reflecting limited plasticity toward this lineage. In contrast, in mouse embryos, Cdx2 is expressed as early as the morula stage, whereas in human embryos, CDX2 expression is initiated at the blastocyst stage [49, 60, 65], suggesting that CDX2 may not be required for the initial specification of trophectoderm in human embryos. Accordingly, alternative strategies have been developed to induce trophectoderm from human PSCs.

Recent studies using human naïve PSCs have enabled efficient induction of trophectoderm‐like cells. In naïve PSCs cultured under PXGL conditions [27], inhibition of MEK/ERK signaling, together with Nodal inhibition, efficiently induces trophectoderm‐like cells [21]. Similarly, naïve PSCs maintained under t2iLGö conditions (MEK/ERK inhibitor, GSK3 inhibitor, hLIF, PKC inhibitor) [35] can be directed toward trophectoderm‐like cells using a combination of MEK/ERK inhibition, Nodal inhibition, JAK inhibition, and BMP4 treatment [22]. The inefficiency of trophectoderm induction in the absence of MEK/ERK inhibition suggests that suppression of this pathway is required for efficient trophectoderm induction. This observation is consistent with the idea that MEK/ERK activity favors alternative lineage trajectories, including hypoblast, whereas its inhibition biases cells toward trophectoderm fate in the context of the plasticity of naïve epiblast and naïve PSCs [16, 21, 23, 54].

In addition, modulation of Hippo–YAP signaling further enhances trophectoderm induction from human naïve PSCs. Inhibition of Hippo signaling promotes nuclear localization of YAP and activation of trophectoderm‐specific transcriptional programs [55, 66]. In human naïve PSCs maintained under PXGL conditions, the tankyrase inhibitor stabilizes Hippo pathway components such as AMOT, thereby restricting YAP nuclear localization [67]. Relief of this inhibition enhances YAP activity and promotes trophectoderm specification. These signaling pathways are also utilized during blastoid formation, where coordinated modulation of MEK/ERK and Hippo–YAP signaling promotes efficient trophectoderm specification.

To dissect lineage‐specific functions more precisely, assembly‐based blastoid systems, in which individual lineages can be independently manipulated, are emerging as an important complement to self‐organizing models. In humans, TSCs resembling cytotrophoblast have been established [68], but long‐term expandable cell lines corresponding to preimplantation trophectoderm have not yet been reported. In contrast, in mice, preimplantation trophectoderm‐like stem cell lines have been established and used to generate assembly‐based blastoids [7]. Establishing comparable human preimplantation trophectoderm cell lines would provide a powerful platform for assembly‐based blastoid systems, enabling precise dissection of lineage‐specific functions and advancing our understanding of human blastocyst development and early post‐implantation processes.

## 4 Applications and Insights from Blastoids

**FIGURE 5:**
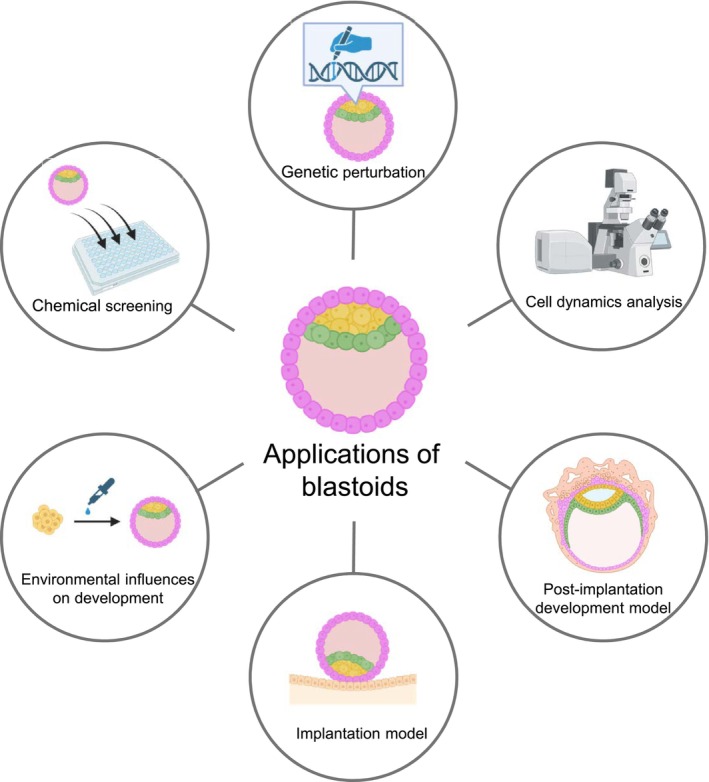
Applications of blastoids in studying early human development. Blastoids enable diverse experimental applications, including genetic perturbation, analysis of cell dynamics, modeling implantation and post‐implantation development, and systematic investigation of environmental and chemical influences on embryogenesis.

Blastoids provide a versatile experimental platform that enables analyses that are difficult or infeasible in natural human embryos (Figure 5). In particular, genetic manipulation and reporter‐based lineage tracing remain limited in human embryos due to ethical and technical constraints, restricting direct functional interrogation of gene regulatory networks and cell dynamics during early development.

A key advantage of blastoids is their ability to recapitulate interactions between embryonic and extraembryonic lineages within a three‐dimensional context. This enables the study of cell–cell interactions and morphogenetic processes that are otherwise difficult to access experimentally. Since their initial report, applications of human blastoids have rapidly expanded, enabling systematic and experimentally tractable investigation of early human development.

### 4.1 Evaluating Environmental Influences on Embryogenesis

Blastoids provide a scalable platform to investigate how external factors influence early human development under controlled conditions. In human blastoids, inhibition of mTOR signaling induces a dormant‐like state, suggesting that diapause‐like regulatory mechanisms may be conserved in humans [69]. These findings highlight how metabolic and signaling cues regulate developmental progression during early embryogenesis. Consistent with this, studies in mouse blastoids have shown that exposure to environmental stressors such as caffeine, ethanol, or nicotine leads to reduced cell numbers and defects in epiblast–primitive endoderm segregation [70]. Together, these results highlight the utility of blastoids for systematically dissecting environmental influences on early embryogenesis.

### 4.2 Dissecting Cellular Dynamics and Molecular Mechanisms

Blastoids provide a powerful platform for dissecting cellular dynamics and molecular mechanisms underlying early human development through imaging and genetic perturbation approaches. In human blastoids, analysis of trophectoderm expansion and ICM division patterns has provided insights into the potential origin of monochorionic twinning [71]. The experimental accessibility of blastoids enables functional interrogation of regulatory elements that are otherwise difficult to study in human embryos. For example, CRISPR‐based perturbation of endogenous retrovirus elements has uncovered a human‐specific transcriptional regulatory layer that shapes epiblast gene expression programs and developmental potential [72].

### 4.3 Blastoids as Models of Implantation

Blastoids provide a platform for modeling human implantation in vitro, a process that remains difficult to study due to limited access to human embryos. Notably, from the earliest studies, human blastoids have been shown to attach to substrates and initiate trophoblast differentiation programs resembling early implantation stages [9, 10, 11].

Traditionally, trophoblast‐derived cancer cell lines such as BeWo [73], JAR [74], and JEG‐3 [75] have been used as in vitro models of implantation. However, these cell lines exhibit chromosomal abnormalities, heterogeneity, and DNA methylation patterns distinct from primary trophoblasts, limiting their physiological relevance [76]. In contrast, human blastoids enable reproducible modeling of trophoblast differentiation from the trophectoderm into syncytiotrophoblast and extravillous trophoblast. In parallel, advances in maternal‐side models have further improved in vitro implantation systems. In addition to classical endometrial cell lines such as Ishikawa [77] and HEC‐1A [78, 79], endometrial organoid‐based platforms have been established [80]. Combining blastoids with endometrial organoids enables analysis of embryo–maternal interactions in a more physiologically relevant context, allowing investigation of implantation dynamics over time [81, 82, 83].

### 4.4 Recapitulating Post‐Implantation‐Like Development

Blastoids offer the potential to explore how early human development can be modeled beyond the blastocyst stage. When cultured in three‐dimensional extracellular matrix environments, human blastoids can exhibit features reminiscent of early post‐implantation development, including primitive streak‐like structures, early lineage diversification associated with gastrulation‐like processes, anterior–posterior axis‐like patterning, and the emergence of primordial germ cell‐like cells [84, 85].

However, the extent to which blastoid‐based systems can robustly and reproducibly model post‐implantation development remains limited. In both human and mouse SCBEMs, more advanced post‐implantation‐like structures have typically been achieved using systems initiated from post‐implantation‐like states rather than blastocyst‐stage models [12, 13, 14, 86, 87].

These observations highlight that model choice among SCBEMs should be guided by the developmental processes of interest. Importantly, evaluating how far blastoids can recapitulate post‐implantation development will be essential for assessing their fidelity to human embryos, while further advances in ex vivo culture systems will be required to extend their developmental potential. Together, these studies establish blastoids as a powerful and experimentally accessible model system. However, it remains essential to rigorously assess how faithfully they recapitulate in vivo human embryogenesis.

## 5 Future Perspectives and Challenges of Blastoids

Blastoids provide a powerful platform for dissecting gene function and spatiotemporal signaling interactions during pre‐implantation and early post‐implantation stages of human development. While genetic manipulation of human embryos and live imaging approaches have begun to emerge [88, 89], these studies remain limited in scope. Blastoids offer a platform that can be further expanded to integrate genome editing, lineage‐specific manipulation, and live imaging approaches, enabling more comprehensive and experimentally tractable investigation of human development. The utility of blastoids is expected to expand further through integration with maternal tissue models, enabling investigation of embryo–maternal interactions and the coordinated regulation of implantation‐related processes and early post‐implantation‐like development, which remain poorly understood in human systems. The incorporation of patient‐derived PSCs may further enable investigation of mechanisms underlying infertility and recurrent pregnancy loss. Blastoids may also serve as a model to investigate the impact of chromosomal abnormalities on early human development. The use of PSCs carrying defined chromosomal alterations or engineered large‐scale genomic modifications [90, 91] enables the generation of mosaic or aneuploid blastoid models, providing a platform to dissect how chromosomal abnormalities influence developmental progression and lineage allocation. In addition, the scalability of blastoid systems enables systematic and high‐throughput investigation of environmental and pharmacological perturbations, with potential applications in understanding infertility, early pregnancy loss, and developmental disorders, as well as informing therapeutic strategies.

Despite these recent advances, current blastoid systems remain limited in their ability to robustly model post‐implantation‐like development. This likely reflects differences from natural blastocysts, including imbalanced lineage composition and divergence in cellular and epigenetic states. These discrepancies may limit the extent to which later developmental processes can be faithfully modeled. Addressing these limitations through improved control of lineage specification and cellular states will be essential for enhancing the fidelity and interpretability of blastoid‐based systems.

As blastoid systems increasingly approximate certain features of natural embryos, careful consideration of ethical boundaries and governance frameworks becomes essential. Blastoids represent a class of SCBEMs and are distinct from embryos formed through fertilization. While blastoids capture key features of early embryos, they remain distinct from natural human blastocysts in their developmental potential and fidelity. Although blastoids have been generated in multiple species, including mice, cows, pigs, and non‐human primates [18, 92, 93, 94, 95], and in some cases transferred into uteri, current evidence indicates that they do not support sustained embryonic development in vivo [18, 92, 94]. According to ISSCR guidelines [96, 97, 98], the transfer of human SCBEMs into a uterus for reproductive purposes is not permitted. Clear definition of research objectives, together with continued co‐development of experimental capabilities and ethical governance, will be essential to ensure responsible and meaningful advancement of the field.

## Funding

This work is supported by the JSPS KAKENHI (24K18047 to H.S., 22K07886 and 25H01350 to A.Y.) and the JST FOREST Program (JPMJFR214Y to A.Y.). A.Y. is supported as a fellow of the Leducq Foundation.

## Ethics Statement

Blastocyst‐like structures (blastoids) were generated by self‐organization of human na*ï*ve PSCs. This research was conducted in accordance with the ISSCR Guidelines, the Guidelines on the Utilization of Human Embryonic Stem Cells, and the Guidelines on the Research on Producing Germ Cells from Human iPS Cells or Human Tissue Stem Cells in Japan. The study was approved by the ethics committees of the University of Tokyo and the Institute of Science Tokyo, as well as by the Ministry of Education, Culture, Sports, Science and Technology, Japan. During the preparation of this work, the authors used ChatGPT (OpenAI) to improve the grammar of the text. The authors reviewed and edited the content and take full responsibility for the final manuscript.

## Conflicts of Interest

The authors declare no conflicts of interest.

## Data Availability

The authors have nothing to report.

## References

[1] A. T. Hertig , J. Rock , and E. C. Adams , “A Description of 34 Human Ova Within the First 17 Days of Development,” American Journal of Anatomy 98 (1956): 435–493. 13362122 10.1002/aja.1000980306

[2] A. T. Hertig , J. Rock , E. C. Adams , and W. J. Mulligan , “On the Preimplanatation Stages of the Human Ovum: A Description of Four Normal and Four Abnormal Specimens Ranging From the Second to the Fifth Day of Development,” Contributions to Embryology 35 (1954): 199–220.

[3] M. J. Evans and M. H. Kaufman , “Establishment in Culture of Pluripotential Cells From Mouse Embryos,” Nature 292 (1981): 154–156. 7242681 10.1038/292154a0

[4] G. R. Martin , “Isolation of a Pluripotent Cell Line From Early Mouse Embryos Cultured in Medium Conditioned by Teratocarcinoma Stem Cells,” Proceedings of the National Academy of Sciences of the United States of America 78 (1981): 7634–7638. 6950406 10.1073/pnas.78.12.7634 PMC349323

[5] J. A. Thomson , J. Itskovitz‐Eldor , S. S. Shapiro , et al., “Embryonic Stem Cell Lines Derived From Human Blastocysts,” Science 282 (1998): 1145–1147. 9804556 10.1126/science.282.5391.1145

[6] S. Tanaka , T. Kunath , A. K. Hadjantonakis , A. Nagy , and J. Rossant , “Promotion of Trophoblast Stem Cell Proliferation by FGF4,” Science 282 (1998): 2072–2075. 9851926 10.1126/science.282.5396.2072

[7] J. Seong , J. Frias‐Aldeguer , V. Holzmann , et al., “Epiblast Inducers Capture Mouse Trophectoderm Stem Cells In Vitro and Pattern Blastoids for Implantation In Utero,” Cell Stem Cell 29 (2022): 1102–1118.e8. 35803228 10.1016/j.stem.2022.06.002

[8] Y. Ohinata , T. A. Endo , H. Sugishita , et al., “Establishment of Mouse Stem Cells That Can Recapitulate the Developmental Potential of Primitive Endoderm,” Science 375 (2022): 574–578. 35113719 10.1126/science.aay3325

[9] L. Yu , Y. Wei , J. Duan , et al., “Blastocyst‐Like Structures Generated From Human Pluripotent Stem Cells,” Nature 591 (2021): 620–626. 33731924 10.1038/s41586-021-03356-y

[10] X. Liu , J. P. Tan , J. Schröder , et al., “Modelling Human Blastocysts by Reprogramming Fibroblasts Into iBlastoids,” Nature 591 (2021): 627–632. 33731926 10.1038/s41586-021-03372-y

[11] A. Yanagida , D. Spindlow , J. Nichols , A. Dattani , A. Smith , and G. Guo , “Naive Stem Cell Blastocyst Model Captures Human Embryo Lineage Segregation,” Cell Stem Cell 28 (2021): 1016–1022.e4. 33957081 10.1016/j.stem.2021.04.031 PMC8189436

[12] B. Oldak , E. Wildschutz , V. Bondarenko , et al., “Complete Human Day 14 Post‐Implantation Embryo Models From Naive ES Cells,” Nature 622 (2023): 562–573. 37673118 10.1038/s41586-023-06604-5 PMC10584686

[13] M. Pedroza , S. I. Gassaloglu , N. Dias , et al., “Self‐Patterning of Human Stem Cells Into Post‐Implantation Lineages,” Nature 622 (2023): 574–583. 37369348 10.1038/s41586-023-06354-4 PMC10584676

[14] B. A. T. Weatherbee , C. W. Gantner , L. K. Iwamoto‐Stohl , et al., “Pluripotent Stem Cell‐Derived Model of the Post‐Implantation Human Embryo,” Nature 622 (2023): 584–593. 37369347 10.1038/s41586-023-06368-y PMC10584688

[15] L. Liu , S. Oura , Z. Markham , et al., “Modeling Post‐Implantation Stages of Human Development Into Early Organogenesis With Stem‐Cell‐Derived Peri‐Gastruloids,” Cell 186 (2023): 3776–3792.e16. 37478861 10.1016/j.cell.2023.07.018

[16] T. Okubo , N. Rivron , M. Kabata , et al., “Hypoblast From Human Pluripotent Stem Cells Regulates Epiblast Development,” Nature 626 (2023): 357–366. 38052228 10.1038/s41586-023-06871-2 PMC10849967

[17] N. Moris , K. Anlas , S. C. van den Brink , et al., “An In Vitro Model of Early Anteroposterior Organization During Human Development,” Nature 582 (2020): 410–415. 32528178 10.1038/s41586-020-2383-9

[18] N. C. Rivron , J. Frias‐Aldeguer , E. J. Vrij , et al., “Blastocyst‐Like Structures Generated Solely From Stem Cells,” Nature 557 (2018): 106–111. 29720634 10.1038/s41586-018-0051-0

[19] X. Gao , M. Nowak‐Imialek , X. Chen , et al., “Establishment of Porcine and Human Expanded Potential Stem Cells,” Nature Cell Biology 21 (2019): 687–699. 31160711 10.1038/s41556-019-0333-2 PMC7035105

[20] Y. Fan , Z. Min , S. Alsolami , et al., “Generation of Human Blastocyst‐Like Structures From Pluripotent Stem Cells,” Cell Discovery 7 (2021): 81. 34489415 10.1038/s41421-021-00316-8 PMC8421367

[21] G. Guo , G. G. Stirparo , S. E. Strawbridge , et al., “Human Naive Epiblast Cells Possess Unrestricted Lineage Potential,” Cell Stem Cell 28 (2021): 1040–1056.e6. 33831366 10.1016/j.stem.2021.02.025 PMC8189439

[22] S. Io , M. Kabata , Y. Iemura , et al., “Capturing Human Trophoblast Development With Naive Pluripotent Stem Cells In Vitro,” Cell Stem Cell 28 (2021): 1023–1039.e13. 33831365 10.1016/j.stem.2021.03.013

[23] A. Dattani , E. Corujo‐Simon , A. Radley , et al., “Naive Pluripotent Stem Cell‐Based Models Capture FGF‐Dependent Human Hypoblast Lineage Specification,” Cell Stem Cell 31 (2024): 1058–1071.e5. 38823388 10.1016/j.stem.2024.05.003

[24] G. Guo , F. von Meyenn , F. Santos , et al., “Naive Pluripotent Stem Cells Derived Directly From Isolated Cells of the Human Inner Cell Mass,” Stem Cell Reports 6 (2016): 437–446. 26947977 10.1016/j.stemcr.2016.02.005 PMC4834040

[25] T. W. Theunissen , B. E. Powell , H. Wang , et al., “Systematic Identification of Culture Conditions for Induction and Maintenance of Naive Human Pluripotency,” Cell Stem Cell 15 (2014): 471–487. 25090446 10.1016/j.stem.2014.07.002 PMC4184977

[26] O. Gafni , L. Weinberger , A. A. Mansour , et al., “Derivation of Novel Human Ground State Naive Pluripotent Stem Cells,” Nature 504 (2013): 282–286. 24172903 10.1038/nature12745

[27] N. Bredenkamp , J. Yang , J. Clarke , et al., “Wnt Inhibition Facilitates RNA‐Mediated Reprogramming of Human Somatic Cells to Naive Pluripotency,” Stem Cell Reports 13 (2019): 1083–1098. 31708477 10.1016/j.stemcr.2019.10.009 PMC6915845

[28] A. Balubaid , S. Alsolami , N. A. Kiani , D. Gomez‐Cabrero , M. Li , and J. Tegner , “A Comparative Analysis of Blastoid Models Through Single‐Cell Transcriptomics,” iScience 27 (2024): 111122. 39524369 10.1016/j.isci.2024.111122 PMC11543915

[29] C. Zhao , A. Plaza Reyes , J. P. Schell , et al., “A Comprehensive Human Embryo Reference Tool Using Single‐Cell RNA‐Sequencing Data,” Nature Methods 22 (2025): 193–206. 39543283 10.1038/s41592-024-02493-2 PMC11725501

[30] J. Nichols and A. Smith , “Naive and Primed Pluripotent States,” Cell Stem Cell 4 (2009): 487–492. 19497275 10.1016/j.stem.2009.05.015

[31] A. Smith , “Formative Pluripotency: The Executive Phase in a Developmental Continuum,” Development 144 (2017): 365–373. 28143843 10.1242/dev.142679 PMC5430734

[32] M. Kinoshita and A. Smith , “Pluripotency Deconstructed,” Development, Growth & Differentiation 60 (2018): 44–52. 10.1111/dgd.12419 29359419

[33] I. G. M. Brons , L. E. Smithers , M. W. B. Trotter , et al., “Derivation of Pluripotent Epiblast Stem Cells From Mammalian Embryos,” Nature 448 (2007): 191–195. 17597762 10.1038/nature05950

[34] P. J. Tesar , J. G. Chenoweth , F. A. Brook , et al., “New Cell Lines From Mouse Epiblast Share Defining Features With Human Embryonic Stem Cells,” Nature 448 (2007): 196–199. 17597760 10.1038/nature05972

[35] Y. Takashima , G. Guo , R. Loos , et al., “Resetting Transcription Factor Control Circuitry Toward Ground‐State Pluripotency in Human,” Cell 158 (2014): 1254–1269. 25215486 10.1016/j.cell.2014.08.029 PMC4162745

[36] K. Cockburn and J. Rossant , “Making the Blastocyst: Lessons From the Mouse,” Journal of Clinical Investigation 120 (2010): 995–1003. 20364097 10.1172/JCI41229 PMC2846056

[37] J. B. Grabarek , K. Zyzyńska , N. Saiz , et al., “Differential Plasticity of Epiblast and Primitive Endoderm Precursors Within the ICM of the Early Mouse Embryo,” Development 139 (2012): 129–139. 22096072 10.1242/dev.067702 PMC3231774

[38] E. Posfai , S. Petropoulos , F. R. O. de Barros , et al., “Position‐ and Hippo Signaling‐Dependent Plasticity During Lineage Segregation in the Early Mouse Embryo,” eLife 6 (2017): e22906. 28226240 10.7554/eLife.22906 PMC5370188

[39] M. Wigger , K. Kisielewska , K. Filimonow , B. Plusa , M. Maleszewski , and A. Suwińska , “Plasticity of the Inner Cell Mass in Mouse Blastocyst Is Restricted by the Activity of FGF/MAPK Pathway,” Scientific Reports 7 (2017): 15136. 29123210 10.1038/s41598-017-15427-0 PMC5680175

[40] C. Chazaud , Y. Yamanaka , T. Pawson , and J. Rossant , “Early Lineage Segregation Between Epiblast and Primitive Endoderm in Mouse Blastocysts Through the Grb2‐MAPK Pathway,” Developmental Cell 10 (2006): 615–624. 16678776 10.1016/j.devcel.2006.02.020

[41] W. A. Pastor , D. Chen , W. Liu , et al., “Naive Human Pluripotent Cells Feature a Methylation Landscape Devoid of Blastocyst or Germline Memory,” Cell Stem Cell 18 (2016): 323–329. 26853856 10.1016/j.stem.2016.01.019 PMC4779431

[42] T. W. Theunissen , M. Friedli , Y. He , et al., “Molecular Criteria for Defining the Naive Human Pluripotent State,” Cell Stem Cell 19 (2016): 502–515. 27424783 10.1016/j.stem.2016.06.011 PMC5065525

[43] J. N. Chousal , R. Morey , S. Srinivasan , et al., “Molecular Profiling of Human Blastocysts Reveals Primitive Endoderm Defects Among Embryos of Decreased Implantation Potential,” Cell Reports 43 (2024): 113701. 38277271 10.1016/j.celrep.2024.113701

[44] X. Wei , X. Fang , X. Yu , et al., “Integrative Analysis of Single‐Cell Embryo Data Reveals Transcriptome Signatures for the Human Pre‐Implantation Inner Cell Mass,” Developmental Biology 502 (2023): 39–49. 37437860 10.1016/j.ydbio.2023.07.004

[45] D. Meistermann , A. Bruneau , S. Loubersac , et al., “Integrated Pseudotime Analysis of Human Pre‐Implantation Embryo Single‐Cell Transcriptomes Reveals the Dynamics of Lineage Specification,” Cell Stem Cell 28 (2021): 1625–1640.e6. 34004179 10.1016/j.stem.2021.04.027

[46] A. Radley , E. Corujo‐Simon , J. Nichols , A. Smith , and S.‐J. Dunn , “Entropy Sorting of Single‐Cell RNA Sequencing Data Reveals the Inner Cell Mass in the Human Pre‐Implantation Embryo,” Stem Cell Reports 18 (2023): 47–63. 36240776 10.1016/j.stemcr.2022.09.007 PMC9859930

[47] A. Radley , S. Boeing , and A. Smith , “Branching Topology of the Human Embryo Transcriptome Revealed by Entropy Sort Feature Weighting,” Development 151 (2024): dev.202832. 10.1242/dev.202832 PMC11213519 38691188

[48] E. W. Kuijk , L. T. A. van Tol , H. Van de Velde , et al., “The Roles of FGF and MAP Kinase Signaling in the Segregation of the Epiblast and Hypoblast Cell Lineages in Bovine and Human Embryos,” Development 139 (2012): 871–882. 22278923 10.1242/dev.071688 PMC3274353

[49] K. K. Niakan and K. Eggan , “Analysis of Human Embryos From Zygote to Blastocyst Reveals Distinct Gene Expression Patterns Relative to the Mouse,” Developmental Biology 375 (2013): 54–64. 23261930 10.1016/j.ydbio.2012.12.008

[50] M. Roode , K. Blair , P. Snell , et al., “Human Hypoblast Formation Is Not Dependent on FGF Signalling,” Developmental Biology 361 (2012): 358–363. 22079695 10.1016/j.ydbio.2011.10.030 PMC3368271

[51] E. Corujo‐Simon , A. H. Radley , and J. Nichols , “Evidence Implicating Sequential Commitment of the Founder Lineages in the Human Blastocyst by Order of Hypoblast Gene Activation,” Development 150 (2023): dev201522. 37102672 10.1242/dev.201522 PMC10233721

[52] J. Nichols , J. Silva , M. Roode , and A. Smith , “Suppression of Erk Signalling Promotes Ground State Pluripotency in the Mouse Embryo,” Development 136 (2009): 3215–3222. 19710168 10.1242/dev.038893 PMC2739140

[53] Y. Yamanaka , F. Lanner , and J. Rossant , “FGF Signal‐Dependent Segregation of Primitive Endoderm and Epiblast in the Mouse Blastocyst,” Development 137 (2010): 715–724. 20147376 10.1242/dev.043471

[54] C. S. Simon , A. McCarthy , L. Woods , et al., “Suppression of ERK Signalling Promotes Pluripotent Epiblast in the Human Blastocyst,” Nature Communications 16 (2025): 6922. 10.1038/s41467-025-61830-x PMC12304225 40721412

[55] H. Kagawa , A. Javali , H. H. Khoei , et al., “Human Blastoids Model Blastocyst Development and Implantation,” Nature 601 (2021): 600–605. 34856602 10.1038/s41586-021-04267-8 PMC8791832

[56] M. Knöfler , S. Haider , L. Saleh , J. Pollheimer , T. K. J. B. Gamage , and J. James , “Human Placenta and Trophoblast Development: Key Molecular Mechanisms and Model Systems,” Cellular and Molecular Life Sciences 76 (2019): 3479–3496. 31049600 10.1007/s00018-019-03104-6 PMC6697717

[57] M. Gauster , G. Moser , S. Wernitznig , N. Kupper , and B. Huppertz , “Early Human Trophoblast Development: From Morphology to Function,” Cellular and Molecular Life Sciences 79 (2022): 345. 35661923 10.1007/s00018-022-04377-0 PMC9167809

[58] C. Chazaud and Y. Yamanaka , “Lineage Specification in the Mouse Preimplantation Embryo,” Development 143 (2016): 1063–1074. 27048685 10.1242/dev.128314

[59] W. J. D. Reeve and C. A. Ziomek , “Distribution of Microvilli on Dissociated Blastomeres From Mouse Embryos: Evidence for Surface Polarization at Compaction,” Development 62 (1981): 339–350. 7276817

[60] D. Strumpf , C.‐A. Mao , Y. Yamanaka , et al., “Cdx2 Is Required for Correct Cell Fate Specification and Differentiation of Trophectoderm in the Mouse Blastocyst,” Development 132 (2005): 2093–2102. 15788452 10.1242/dev.01801

[61] R. Yagi , M. J. Kohn , I. Karavanova , et al., “Transcription Factor TEAD4 Specifies the Trophectoderm Lineage at the Beginning of Mammalian Development,” Development 134 (2007): 3827–3836. 17913785 10.1242/dev.010223

[62] N. Nishioka , K.‐I. Inoue , K. Adachi , et al., “The Hippo Signaling Pathway Components Lats and Yap Pattern Tead4 Activity to Distinguish Mouse Trophectoderm From Inner Cell Mass,” Developmental Cell 16 (2009): 398–410. 19289085 10.1016/j.devcel.2009.02.003

[63] C. Gerri , A. McCarthy , G. Alanis‐Lobato , et al., “Initiation of a Conserved Trophectoderm Program in Human, Cow and Mouse Embryos,” Nature 587 (2020): 443–447. 32968278 10.1038/s41586-020-2759-x PMC7116563

[64] M. Zhu , M. Shahbazi , A. Martin , et al., “Human Embryo Polarization Requires PLC Signaling to Mediate Trophectoderm Specification,” eLife 10 (2021): e65068. 34569938 10.7554/eLife.65068 PMC8514238

[65] H. Niwa , Y. Toyooka , D. Shimosato , et al., “Interaction Between Oct3/4 and Cdx2 Determines Trophectoderm Differentiation,” Cell 123 (2005): 917–929. 16325584 10.1016/j.cell.2005.08.040

[66] L. Yu , D. Logsdon , C. A. Pinzon‐Arteaga , et al., “Large‐Scale Production of Human Blastoids Amenable to Modeling Blastocyst Development and Maternal‐Fetal Cross Talk,” Cell Stem Cell 30 (2023): 1246–1261.e9. 37683605 10.1016/j.stem.2023.08.002

[67] A. Dattani , T. Huang , C. Liddle , A. Smith , and G. Guo , “Suppression of YAP Safeguards Human Naïve Pluripotency,” Development 149 (2022): dev200988. 36398796 10.1242/dev.200988 PMC9845734

[68] H. Okae , H. Toh , T. Sato , et al., “Derivation of Human Trophoblast Stem Cells,” Cell Stem Cell 22 (2018): 50–63.e6. 29249463 10.1016/j.stem.2017.11.004

[69] D. P. Iyer , H. H. Khoei , V. A. van der Weijden , et al., “mTOR Activity Paces Human Blastocyst Stage Developmental Progression,” Cell 187 (2024): 6566–6583.e22. 39332412 10.1016/j.cell.2024.08.048 PMC7617234

[70] V. Jorgensen , M. Bao , S. Junyent , et al., “Efficient Stem Cell‐Derived Mouse Embryo Models for Environmental Studies,” Developmental Cell 61 (2026): 193–207.e6. 40882624 10.1016/j.devcel.2025.08.004 PMC13197000

[71] D. G. Luijkx , A. Ak , G. Guo , C. A. van Blitterswijk , S. Giselbrecht , and E. J. Vrij , “Monochorionic Twinning in Bioengineered Human Embryo Models,” Advanced Materials 36 (2024): e2313306. 38593372 10.1002/adma.202313306

[72] R. Fueyo , S. Wang , O. J. Crocker , T. Swigut , H. Nakauchi , and J. Wysocka , “A Human‐Specific Regulatory Mechanism Revealed in a Pre‐Implantation Model,” Nature 647 (2025): 238–247. 41034587 10.1038/s41586-025-09571-1 PMC12589118

[73] R. A. Pattillo and G. O. Gey , “The Establishment of a Cell Line of Human Hormone‐Synthesizing Trophoblastic Cells In Vitro,” Cancer Research 28 (1968): 1231–1236. 4299001

[74] R. Pattillo , A. Ruckert , R. Hussa , R. Bernstein , and E. Delfs , “The JAr Cell Line ‐ Continuous Human Multi‐Hormone Production and Controls,” In Vitro Cellular & Developmental Biology. Plant 6 (1971): 398–399.

[75] P. O. Kohler and W. E. Bridson , “Isolation of Hormone‐Producing Clonal Lines of Human Choriocarcinoma,” Journal of Clinical Endocrinology and Metabolism 32 (1971): 683–687. 5103722 10.1210/jcem-32-5-683

[76] B. Novakovic , L. Gordon , N. C. Wong , et al., “Wide‐Ranging DNA Methylation Differences of Primary Trophoblast Cell Populations and Derived Cell Lines: Implications and Opportunities for Understanding Trophoblast Function,” Molecular Human Reproduction 17 (2011): 344–353. 21289002 10.1093/molehr/gar005 PMC3797416

[77] M. Nishida , K. Kasahara , M. Kaneko , H. Iwasaki , and K. Hayashi , “Establishment of a New Human Endometrial Adenocarcinoma Cell Line, Ishikawa Cells, Containing Estrogen and Progesterone Receptors,” Nihon Sanka Fujinka Gakkai Zasshi 37 (1985): 1103–1111. 4031568

[78] H. Kuramoto , S. Tamura , and Y. Notake , “Establishment of a Cell Line of Human Endometrial Adenocarcinoma In Vitro,” American Journal of Obstetrics and Gynecology 114 (1972): 1012–1019. 4673779 10.1016/0002-9378(72)90861-7

[79] H. Kuramoto , “Studies of the Growth and Cytogenetic Properties of Human Endometrial Adenocarcinoma in Culture and Its Development Into an Established Line,” Acta Obstetrica et Gynaecologica Japonica 19 (1972): 47–58. 4678779

[80] M. Y. Turco , L. Gardner , J. Hughes , et al., “Long‐Term, Hormone‐Responsive Organoid Cultures of Human Endometrium in a Chemically Defined Medium,” Nature Cell Biology 19 (2017): 568–577. 28394884 10.1038/ncb3516 PMC5410172

[81] S. Shibata , S. Endo , L. A. E. Nagai , et al., “Modeling Embryo‐Endometrial Interface Recapitulating Human Embryo Implantation,” Science Advances 10 (2024): eadi4819. 38394208 10.1126/sciadv.adi4819 PMC10889356

[82] Q. Li , Y. Yuan , W. Zhao , et al., “A 3D In Vitro Model for Studying Human Implantation and Implantation Failure,” Cell 189 (2026): 70–86.e20. 41443192 10.1016/j.cell.2025.10.026

[83] M. A. Molè , S. Elderkin , I. Zorzan , et al., “Modeling Human Embryo Implantation In Vitro,” Cell 189 (2026): 87–105.e28. 41443191 10.1016/j.cell.2025.10.027

[84] R. M. Karvas , J. E. Zemke , S. S. Ali , et al., “3D‐Cultured Blastoids Model Human Embryogenesis From Pre‐Implantation to Early Gastrulation Stages,” Cell Stem Cell 30 (2023): 1148–1165.e7. 37683602 10.1016/j.stem.2023.08.005

[85] H. Xie , C. An , B. Bai , et al., “Modeling Early Gastrulation in Human Blastoids With DNA Methylation Patterns of Natural Blastocysts,” Cell Stem Cell 32 (2025): 409–425.e8. 39814012 10.1016/j.stem.2024.12.010

[86] S. Tarazi , A. Aguilera‐Castrejon , C. Joubran , et al., “Post‐Gastrulation Synthetic Embryos Generated ex Utero From Mouse Naive ESCs,” Cell 185 (2022): 3290–3306.e25. 35988542 10.1016/j.cell.2022.07.028 PMC9439721

[87] G. Amadei , C. E. Handford , C. Qiu , et al., “Synthetic Embryos Complete Gastrulation to Neurulation and Organogenesis,” Nature 610 (2022): 1–3. 10.1038/s41586-022-05246-3 PMC9534772 36007540

[88] N. M. E. Fogarty , A. McCarthy , K. E. Snijders , et al., “Genome Editing Reveals a Role for OCT4 in Human Embryogenesis,” Nature 550 (2017): 67–73. 28953884 10.1038/nature24033 PMC5815497

[89] A. Domingo‐Muelas , R. M. Skory , A. A. Moverley , et al., “Human Embryo Live Imaging Reveals Nuclear DNA Shedding During Blastocyst Expansion and Biopsy,” Cell 186 (2023): 3166–3181.e18. 37413989 10.1016/j.cell.2023.06.003 PMC11170958

[90] Y. Kazuki and M. Oshimura , “Human Artificial Chromosomes for Gene Delivery and the Development of Animal Models,” Molecular Therapy 19 (2011): 1591–1601. 21750534 10.1038/mt.2011.136 PMC3182354

[91] N. Uno , S. Abe , M. Oshimura , and Y. Kazuki , “Combinations of Chromosome Transfer and Genome Editing for the Development of Cell/Animal Models of Human Disease and Humanized Animal Models,” Journal of Human Genetics 63 (2018): 145–156. 29180645 10.1038/s10038-017-0378-7

[92] C. A. Pinzón‐Arteaga , Y. Wang , Y. Wei , et al., “Bovine Blastocyst‐Like Structures Derived From Stem Cell Cultures,” Cell Stem Cell 30 (2023): 611–616.e7. 37146582 10.1016/j.stem.2023.04.003 PMC10230549

[93] J. Xiang , H. Wang , B. Shi , et al., “Pig Blastocyst‐Like Structure Models From Embryonic Stem Cells,” Cell Discovery 10 (2024): 72. 38956027 10.1038/s41421-024-00693-w PMC11219778

[94] J. Li , Q. Zhu , J. Cao , et al., “Cynomolgus Monkey Embryo Model Captures Gastrulation and Early Pregnancy,” Cell Stem Cell 30 (2023): 362–377.e7. 37028403 10.1016/j.stem.2023.03.009

[95] T. Huang , A. Radley , A. Yanagida , et al., “Inhibition of PRC2 Enables Self‐Renewal of Blastoid‐Competent Naive Pluripotent Stem Cells From Chimpanzee,” Cell Stem Cell 32 (2025): 627–639.e8. 40015279 10.1016/j.stem.2025.02.002 PMC7617839

[96] R. Lovell‐Badge , E. Anthony , R. A. Barker , et al., “ISSCR Guidelines for Stem Cell Research and Clinical Translation: The 2021 Update,” Stem Cell Reports 16 (2021): 1398–1408. 34048692 10.1016/j.stemcr.2021.05.012 PMC8190668

[97] A. T. Clark , A. Brivanlou , J. Fu , et al., “Human Embryo Research, Stem Cell‐Derived Embryo Models and In Vitro Gametogenesis: Considerations Leading to the Revised ISSCR Guidelines,” Stem Cell Reports 16 (2021): 1416–1424. 34048690 10.1016/j.stemcr.2021.05.008 PMC8190666

[98] A. T. Clark , H. Cook‐Andersen , S. Franklin , et al., “Stem Cell‐Based Embryo Models: The 2021 ISSCR Stem Cell Guidelines Revisited,” Stem Cell Reports 20 (2025): 102514. 40499509 10.1016/j.stemcr.2025.102514 PMC12181966

